# Endocytic turnover of Rab8 controls cell polarization

**DOI:** 10.1242/jcs.195420

**Published:** 2017-03-15

**Authors:** Maite Vidal-Quadras, Mikkel R. Holst, Monika K. Francis, Elin Larsson, Mariam Hachimi, Wai-Lok Yau, Johan Peränen, Fernando Martín-Belmonte, Richard Lundmark

**Affiliations:** 1 Integrative Medical Biology, Umeå University, Umeå 901 87, Sweden; 2 Medical Biochemistry and Biophysics, Laboratory for Molecular Infection Medicine Sweden, Umeå University, Umeå 901 87, Sweden; 3 Centro de Biología Molecular Severo Ochoa, Consejo Superior de Investigaciones Científicas-UAM, Madrid 28049, Spain; 4 Department of Anatomy, Faculty of Medicine, University of Helsinki, Helsinki FIN-00014, Finland

**Keywords:** Rab8, GRAF1, Cell polarization, Endocytosis

## Abstract

Adaptation of cell shape and polarization through the formation and retraction of cellular protrusions requires balancing of endocytosis and exocytosis combined with fine-tuning of the local activity of small GTPases like Rab8. Here, we show that endocytic turnover of the plasma membrane at protrusions is directly coupled to surface removal and inactivation of Rab8. Removal is induced by reduced membrane tension and mediated by the GTPase regulator associated with focal adhesion kinase-1 (GRAF1, also known as ARHGAP26), a regulator of clathrin-independent endocytosis. GRAF1-depleted cells were deficient in multi-directional spreading and displayed elevated levels of GTP-loaded Rab8, which was accumulated at the tips of static protrusions. Furthermore, GRAF1 depletion impaired lumen formation and spindle orientation in a 3D cell culture system, indicating that GRAF1 activity regulates polarity establishment. Our data suggest that GRAF1-mediated removal of Rab8 from the cell surface restricts its activity during protrusion formation, thereby facilitating dynamic adjustment of the polarity axis.

## Abstract (precis)

**Highlighted Article:** GRAF1 mediates inactivation and endocytic removal of Rab8 from cell surface protrusions, thereby facilitating dynamic adjustment of the polarity axis.

## INTRODUCTION

Polarity establishment or breaking of symmetry is essential for migration, differentiation and cell morphogenesis. Polarization is driven by molecular adaptation in response to extracellular cues and the membrane tension, which leads to the establishment of a polarity axis. The Rho-family GTPase Cdc42 has a central role in polarity establishment from yeast to humans, and its localization and activity segregates spontaneously even in absence of upstream spatial cues. In epithelial cells, like MDCK cells, the apical localization of Cdc42 is crucial in order to define the apico-basal axis (Martin-Belmonte et al., 2007). The active GTP-bound form of Cdc42 interacts with the PAR complex, comprised of Par6 (of which mammals have several isoforms) and atypical protein kinase C (aPKC), at the apical plasma membrane (Garrard et al., 2003; Johansson et al., 2000; Lin et al., 2000). Cdc42, together with the Par complex, reaches the apical membrane in a Rab8-dependent manner during epithelial lumen formation (Bryant et al., 2010; Hattula et al., 2006; Sato et al., 2007). Thus, Rab8 (which has two isoforms, Rab8a and Rab8b) is an important regulator of polarized trafficking that enables protrusion formation, ciliogenesis or intestinal epithelial formation (Hattula et al., 2006; Nachury et al., 2007; Sakamori et al., 2012). Cdc42 and Rab8 belong to the family of small G-proteins that, in their GTP-bound state, interact with various types of effector molecules leading to downstream effects. GTP-loading and hydrolysis is stimulated by guanidine exchange factors (GEFs) and GTPase-activating proteins (GAPs), respectively, which regulate the activity of the GTPases. Cdc42 and Rab8 anchor to membranes via a C-terminal prenylation, and the local activity of Cdc42 and Rab8 needs to be dynamically maintained through cycling between the surface and internal pools (Freisinger et al., 2013). In yeast, endocytosis has been shown to localize Cdc42 to vesicles positive for Sec-4 (the homolog of Rab8), as a positive loop to bring Cdc42 back to the plasma membrane (Balklava et al., 2007; Watson et al., 2014).

Several distinct pathways have been proposed to facilitate endocytic turnover at polarized cell regions; one of these, the clathrin-independent carrier (CLIC) pathway, is responsible for bulk endocytosis at the leading edge (Howes et al., 2010). Cdc42 and GTPase regulator associated with focal adhesion kinase-1 (GRAF1, also known as ARHGAP26) are regulators of this pathway (Lundmark et al., 2008; Sabharanjak et al., 2002). The multidomain protein GRAF1 is composed of a Bin, amphiphysin RVS161/167 (BAR), a pleckstrin homology (PH), a GAP and Src homology (SH3) domain. The BAR and PH domains can generate and/or stabilize highly curved endocytic membranes (Lundmark et al., 2008) and the GAP domain is active against Cdc42 and RhoA (Hildebrand et al., 1996; Jelen et al., 2009; Longenecker et al., 2000). Depletion of either GRAF1 or Cdc42 leads to a substantial reduction in fluid uptake and impaired cell spreading (Doherty et al., 2011; Francis et al., 2015). However, it remains to be elucidated how the vesicular trafficking and local activity of small GTPases are coordinated during endocytic membrane turnover at cellular protrusions.

In this study, we demonstrate that GRAF1 facilitates cell surface removal and inactivation of Rab8 through clathrin-independent endocytosis. Impairment of this process leads to elevated levels of GTP-loaded Rab8 and an inability of cells to adjust the polarity axis. Based on these data, we propose that GRAF1-mediated removal of Rab8 from the cell is important for balancing membrane redistribution between growing and retracting regions of the cell, thereby supporting polarity establishment.

## RESULTS

### Active Rab8 is removed from cell surface protrusions via GRAF1-dependent endocytosis

**Fig. 1.**
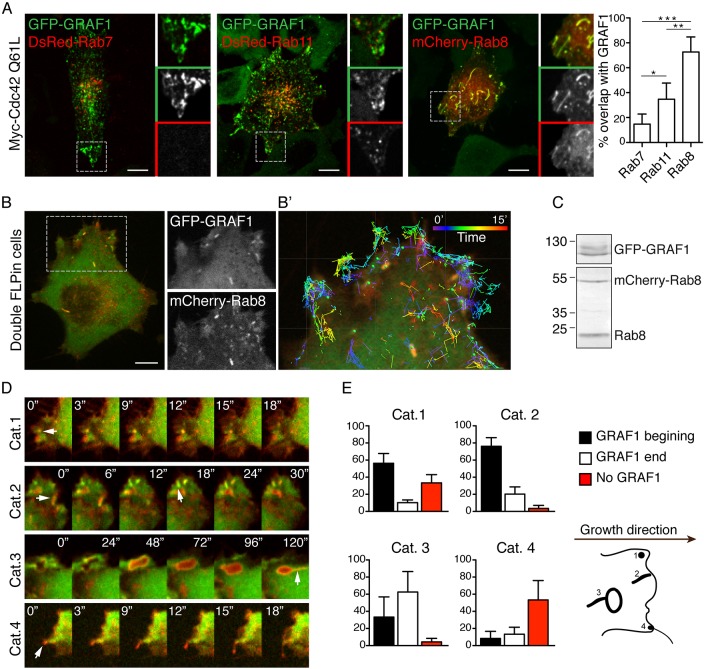
**Rab8 is present in GRAF1 carriers at cellular protrusions.** (A) Fluorescent micrographs from live-cell imaging of Flp-In T-REx HeLa cells expressing GFP–GRAF1 co-transfected with Myc–Cdc42Q61L and the indicated Rab protein. The bar plot to the right shows a quantification (mean±s.d., *n*=3 independent experiments) of the overlap between GRAF1-positive structures and different Rab-positive structures as indicated. *P≤0.05, **P≤0.01, ***P≤0.001 (Kruskal–Wallis test). (B) Fluorescent micrographs from live-cell imaging of the double Flp-In T-REx HeLa cells expressing GFP–GRAF1 (green) and mCherry–Rab8 (red). (B′) Magnification of the cell protrusion with superimposed tracks of structures positive for both proteins; tracks were color coded to show that structures positive for GRAF1 and Rab8 that occurred during the 15-min acquisition. (C) Immunoblot analysis of cell lysate from the double Flp-In cells. GFP–GRAF1 was detected with anti-GRAF1 and mCherry–Rab8 and Rab8 endogenous were detected with anti-Rab8 antibodies. (D) Image sequence captured using live-cell microscopy of the double Flp-In T-REx HeLa cell line showing examples of structures that appear at growing protrusions classified into four categories (arrows). (E) Quantification of the percentage of GRAF1 presence within the different categories. Bar graphs in E represent mean±s.e.m. of 55 protrusions from 30 cells. Scale bar: 10 μm.

Expression of the constitutively active mutant of Cdc42 (Cdc42Q61L) has been shown to stall internalized GRAF1-positive vesicles (Francis et al., 2015), providing a tool to identify additional components involved in the CLIC pathway. Since the stalled vesicles were negative for Rab5 (Francis et al., 2015), we wanted to investigate whether other members of the Rab family were involved in this pathway. We transiently co-transfected GFP–GRAF1 Flp-In T-REx HeLa cells with Myc–Cdc42Q61L and DsRed–Rab7, mCherry–Rab8 or DsRed–Rab11 (Rab7a, Rab8a and Rab11a isoforms). Spinning disk confocal live-cell imaging revealed extensive colocalization between Rab8 and the characteristic GRAF1-positive membrane structures induced by the expression of Myc–Cdc42Q61L (Fig. 1A). Although DsRed–Rab7 and DsRed–Rab11 were detected in vesicular structures, these did not overlap with GRAF1 to the same extent. GFP–GRAF1 also colocalized with endogenous Rab8 and with mCherry–Rab8 in absence of Myc–Cdc42Q61L, showing that mutant Cdc42 is not required for colocalization of GRAF1 and Rab8 (Fig. S1A,B). By assessing colocalization of the constitutively active mutant mCherry–Rab8Q67L, or the Rab8 activity probe MICAL-L1-CT (Kobayashi et al., 2014), we found that GFP–GRAF1 colocalized with the active form of Rab8 (Fig. S1C,D). However, no colocalization was observed between GRAF1 and the constitutively inactive mutant mCherry–Rab8T22N (Fig. S1C). In order to investigate whether Rab8 was in close proximity to GRAF1, we used a Flp-In T-REx HeLa cell line expressing APEX-tagged GRAF1, where APEX-mediated biotinylation of neighboring proteins can be induced by addition of biotin phenol to the cells. When Myc–Cdc42Q61L was coexpressed in these cells, we were indeed able to pull down biotinylated Rab8 with streptavidin beads. GRAF1 and Cdc42 were also pulled down and, in addition, EHD1 and MICAL-L1 were identified using this approach (Fig. S1E). MICAL-L1 has been shown to interact with both GRAF1 and Rab8, and our data suggest that these proteins might form a complex on membranes as previously shown (Cai et al., 2014).

To study the interplay between GRAF1 and Rab8, we created a double Flp-In T-REx HeLa cell line that simultaneously expresses GFP–GRAF1 and mCherry–Rab8 at equimolar levels following induction with doxycycline (Fig. 1C). Live imaging and tracking revealed extensive colocalization of both proteins in dynamic structures at cellular protrusions in these cells (Fig. 1B; Movie 1). Rab8 has been observed to localize to both exocytic and endocytic membrane carriers (Hattula et al., 2006). Therefore, to address the specific role of GRAF1, we classified Rab8-positive structures into four categories based on their morphology and behavior (Fig. 1D,E). Category 1 and 2 correspond to small round or tubular structures, respectively, that formed from the cell edge. Category 3 represents larger macropinosome-like structures, and category 4 corresponds to vesicular structures that travelled towards the cell edge and disappeared (indicative of exocytosis) (Fig. 1D). Quantification showed that the structures in categories 1 and 2 were already positive for GFP–GRAF1 at the time of their initial appearance, and that GFP–GRAF1 recruitment led to the generation of tubules from pre-existing macropinosome-like structures. However, GFP–GRAF1 was not present in the mCherry–Rab8 vesicles in category 4. This suggested that GFP–GRAF1 was involved in endocytic but not exocytic Rab8-positive trafficking events. In agreement with this, we observed the formation of tubular structures from the cell surface that were positive for GFP–GRAF1, mCherry–Rab8 and the endocytic marker cholera toxin (CTxB) (Fig. S1F). However, Rab8, but not GFP–GRAF1, colocalized with the PH domain of FAPP1 (BFP–FAPP1-PH), which binds to phosphatidylinositol 4-phosphate and Arf1 and marks Golgi-derived secretory vesicles (Balla et al., 2005; Levine and Munro, 2002) (Fig. S1G). To test whether Rab8 was a prerequisite for endocytic membrane turnover at protrusions, HeLa cells depleted of Rab8 or GRAF1 were allowed to internalize fluorescent dextran and were analyzed by microscopy. Rab8 depletion did not affect the dynamic assembly of GFP–GRAF1 at protrusions or the characteristic GRAF1-dependent uptake of dextran at protrusions (Fig. S1H,I), suggesting that Rab8 is not required for the formation of CLICs.

### Reduced membrane tension induces endocytosis of surface-associated Rab8 together with GRAF1

**Fig. 2.**
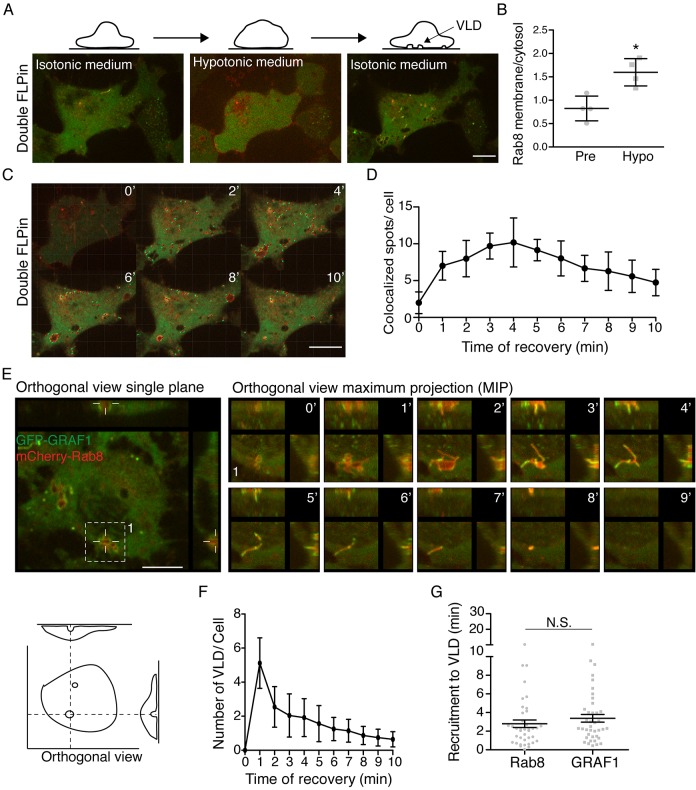
**Changes in membrane tension regulate the membrane localization of GRAF1.** (A) Representative images from a live-cell movie of double Flp-In T-REx HeLa cells during the different steps of the experiment. The cartoon on top illustrates the cellular morphology in the different phases. (B) Quantification of the ratio of Rab8 localized in the membrane. Rab8 intensity was measured at the plasma membrane and in the cytosol before and during the hypotonic treatment. The mean±s.d. from four independent experiments, where at least two cells per experiment were analyzed, is shown. (C) Image sequence of the recovery after hypotonic treatment showing GFP–GRAF1 (green) and mCherry–Rab8 (red). Colocalized spots from the IMARIS segmentation of the channels are superimposed. (D) Quantification (mean±s.d.) of colocalized spots over time from four independent experiments. (E) Orthogonal view of a cell highlighting a VLD. The right panel shows the maximum projection over time of the VLD indicated by the dashed square. A representation of the orthogonal view used to determine the appearance and clearance of VLDs during the recovery phase is shown in the lower panel. (F) Quantification of the number of VLDs observed during recovery from hypotonic treatment per cell from four independent experiments (mean±s.d.; *n*=45 cells). (G) Quantification of recruitment of Rab8 and GRAF1 to VLDs from four different experiments as described in the Materials and Methods (mean±s.e.m.; *n*=40 VLDs). Scale bar: 10 μm. *P≤0.05; N.S., not significant (*t*-test).

Alterations in the membrane tension at cell protrusions are thought to determine the dynamic ratio between endocytosis and exocytosis (Houk et al., 2012; Lieber et al., 2015). Cell swelling increases membrane tension and induces exocytosis, while shrinking of the cell volume decreases tension and induces endocytosis (Kosmalska et al., 2015; Raucher and Sheetz, 2000). To analyze how Rab8 and GRAF1 responded to such changes, double Flp-In T-REx HeLa cells were seeded in microfluidic plates to control the timing of the medium exchange during the time lapse acquired by spinning disk confocal microscopy. First, cells were imaged with the normal culture medium (isotonic medium) for 2 min, then, the medium was changed to a hypotonic medium to swell the cells and increase the membrane tension (Fig. 2A). In response to hypotonic treatment, the relative plasma membrane localization of mCherry–Rab8 was increased while GFP–GRAF1 was rendered completely cytosolic (Fig. 2B; Movie 2). This is consistent with inhibition of endocytosis upon increased membrane tension and the role of Rab8 in exocytic vesicle fusion to add membrane (Boulant et al., 2011; Mavor et al., 2016). However, when the hypotonic medium was changed to isotonic medium (recovery), we observed a striking appearance of GFP–GRAF1- and mCherry–Rab8-coated vesicles and tubules (Fig. 2A). Tracking of structures positive for both proteins (colocalized spots), showed that the number of colocalized spots reached a maximum after 4 min of recovery (Fig. 2C,D), suggesting that the decrease in membrane tension induced a rapid and transient burst in GRAF1-mediated endocytosis of mCherry-Rab8. Interestingly, we also observed the immediate appearance of vacuole-like dilatations (VLDs), which are large invaginations caused by a mechanical response when secreted water is entrapped between the membrane and the glass surface (Fig. 2E) (Gauthier et al., 2012; Reuzeau et al., 1995). Quantification of the number of VLDs per cell revealed a maximum at 1 min after exchange to isotonic medium and that the number was reduced during 10 min of recovery (Fig. 2F). Clearance of VLDs has been shown to depend on an unknown energy-dependent process (Kosmalska et al., 2015). We analyzed the recruitment of mCherry–Rab8 and GFP–GRAF1 to VLDs and found that both proteins were recruited at ∼3 min after the VLDs appeared (Fig. 2G). Time lapse of the maximum projection from the orthogonal view clearly showed that the mCherry–Rab8-positive VLDs were transformed into tubular structures that were decorated by GFP–GRAF1 before they disappeared (Fig. 2E; Movie 3). We also found that GTP-bound, but not GDP-bound, Rab8 was recruited to VLDs, as determined by assessing the localization of the constitutively inactive (T22N) and constitutively active (Q67L) Rab8 mutants (Fig. S2A). These data suggest that the mechanically induced VLDs acquired GTP-bound Rab8 and GRAF1, which induced endocytosis of the VLD membrane. Based on our results, we propose that membrane tension dictates the balance between CLIC-mediated internalization of Rab8 and fusion of Rab8-positive vesicles at protrusions.

### GRAF1 regulates the levels of GTP-bound Rab8

**Fig. 3.**
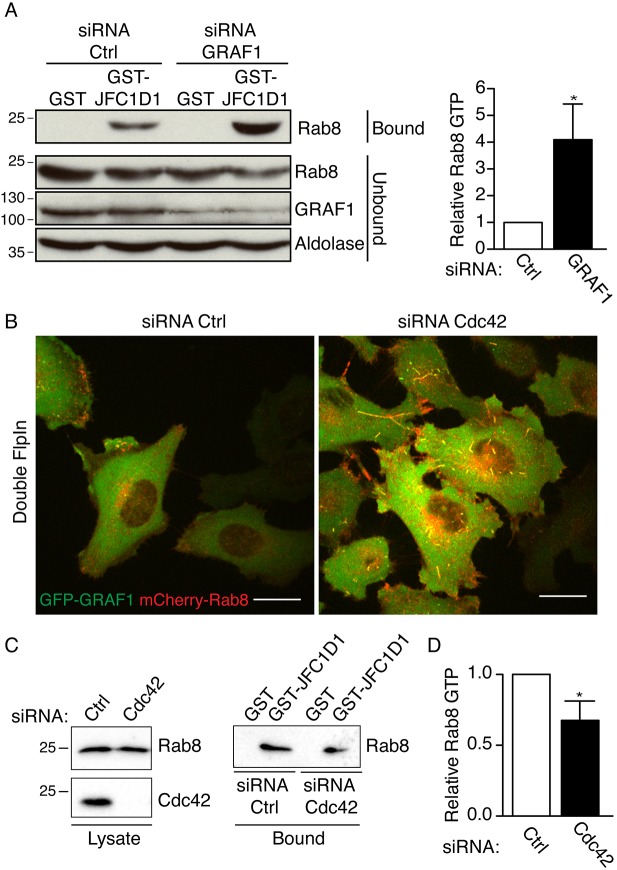
**GRAF1 regulates Rab8 activity.** (A) Immunoblot analysis of a Rab8 activity pulldown assay. Lysates from GFP-GRAF1 Flp-In T-REx HeLa cells transfected with control siRNA (Ctrl) or siRNA against GRAF1 were incubated with GST–JFC1D1 or GST to pulldown GTP-bound Rab8 or GST, as a negative control. The right panel shows the quantification (mean±s.d.) of three independent experiments. (B) Micrograph of double Flp-In T-REx HeLa cells expressing GFP–GRAF1 (green) and mCherry–Rab8 (red) and transfected with control siRNA (Ctrl) or siRNA against Cdc42. Scale bar: 10 μm. (C) Immunoblot analysis of a Rab8 activity pulldown assay. Lysates from GFP-GRAF1 Flp-In T-REx HeLa cells transfected with control siRNA (Ctrl) or siRNA against Cdc42 were incubated with GST–JFC1D1 or GST to pulldown GTP-bound Rab8 or GST, as a negative control. (D) Quantification of Rab8 activity from four independent experiments performed as in C. *P≤0.05 (*t*-test).

It has been suggested that Rab8 is inactivated through GTP hydrolysis following internalization (Hokanson and Bretscher, 2012). To determine whether GRAF1 influenced the nucleotide-bound state of Rab8, we used GST–JFC1D1 (Hattula et al., 2006) to pull down active Rab8 from control cells and cells depleted of GRAF1 (Fig. 3A). GRAF1 depletion led to a 4-fold increase in the levels of GTP-bound Rab8 compared to control (Fig. 3A). This indicated that the tension-driven internalization of Rab8 mediated by GRAF1 was regulating the activity of Rab8. Depletion of Cdc42 results in reduced membrane tension (Bretou et al., 2014), but blocks CLIC-dependent uptake and induces long surface-connected tubules decorated by GRAF1 (Francis et al., 2015). To test whether endocytosis per se was important for Rab8 inactivation, we depleted cells of Cdc42. Activity assays revealed that the levels of GTP-bound Rab8 were decreased following Cdc42 depletion, and micrographs showed that mCherry–Rab8 was trapped in the long GFP–GRAF1-decorated tubules (Fig. 3B–D). These data indicate that the tension-induced spatial segregation of Rab8 into GRAF1-positive membrane tubules is sufficient to promote inactivation, but that endocytosis of these tubules is not required.

### Rab8 and MT1-MMP are highly polarized in GRAF1-depleted cells

**Fig. 4.**
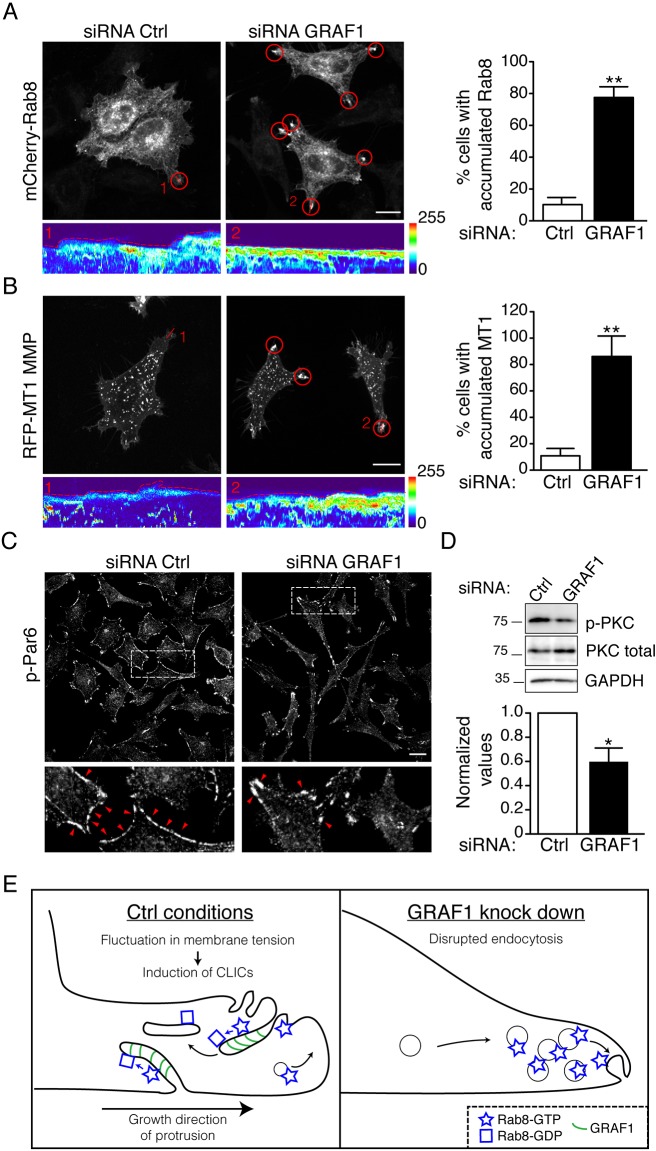
**GRAF1 depletion alters Rab8-dependent trafficking.** (A,B) Representative micrographs from 5-min movies of mCherry-Rab8 Flp-In T-REx HeLa cells transfected with indicated siRNAs (A) and GFP-GRAF1 Flp-In T-REx HeLa cells transfected with MT1-MMP–mRFP (B). Red circles indicate mCherry–Rab8 (A) or MT1-MMP–mRFP (B) accumulation at the tip. Kymographs are taken from the indicated lines and the pseudo color represents localized fluorescence intensity. The dotted line illustrates the cell boundary. Right panels show quantifications (mean±s.d.) from three independent experiments. (C) p-Par6 staining in Flp-In T-REx HeLa cells transfected with indicated siRNA, red arrowheads illustrate loss of p-Par6 staining in the cell periphery after GRAF1 depletion. (D) Immunoblot analysis of phosphorylated aPKC (p-PKC) and total aPKC from lysates of GFP-GRAF1 Flp-In T-REx HeLa cells transfected with Ctrl or GRAF1 siRNA. Western blots from four independent experiments were quantified and normalized to the levels of total aPKC and GAPDH (mean±s.d.). *P≤0.05; **P≤0.01 (*t*-test). Scale bars: 10 μm. (E) Cartoon model illustrating the localization of GRAF1 and Rab8 in control conditions and after GRAF1 depletion. In cells lacking GRAF1, Rab8 is not removed from the cell surface and inactivated, which will increase total Rab8 activity and consequently, enhance Rab8-dependent transport to the plasma membrane.

To visualize how GRAF1 depletion affected Rab8 trafficking, live-cell microscopy was performed on a mCherry-Rab8 Flp-In T-REx HeLa cell line transfected with control or GRAF1 siRNA. Interestingly, we found that Rab8 was accumulated distinctively at the tip of more static protrusions in the GRAF1-depleted cells (Fig. 4A). The accumulated intensity of Rab8 was detected both as mobile vesicles enriched at the protrusions and in larger stable structures that appeared continuous with the plasma membrane (kymographs, Fig. 4A). To test whether secretory vesicles containing Rab8 were mobilized to these regions, we analyzed the localization of MT1-MMP–mRFP (MT1-MMP is also known as MMP14), a cargo in the Rab8-dependent secretory pathway (Bravo-Cordero et al., 2007). Consistent with the Rab8 localization, MT1-MMP–mRFP was also enriched at the protrusion tips (Fig. 4B). Quantification showed that 80% of the cells displayed this phenotype. Additionally, phosphorylated Par6 (p-Par6; the Par6a isoform encoded by *PARD6A*) showed a similar type of asymmetric redistribution in GRAF1-depleted cells (Fig. 4C). In comparison, p-Par6 was detected along the entire membrane in control cells. This is in agreement with the finding that Par6 is trafficked by Rab8-positive vesicles to form the polarity complex together with Cdc42 and aPKC (Bryant et al., 2010). In parallel, we analyzed the levels of phosphorylated aPKC, because it is responsible for Par6 phosphorylation (Fig. 4D) (Gunaratne et al., 2013). We found a significant decrease in the levels of pPKC in GRAF1-depleted cells, which is coherent with the loss of the uniform plasma membrane stain of p-Par6. However, analysis of the Cdc42 activity using the Rac/Cdc42-binding domain (PBD) of the human p21 activated kinase 1 protein (PAK) coupled to beads (PAK-PDB) showed that the global levels of GTP-bound Cdc42 were not significantly changed following GRAF1 or Rab8 knockdown (Fig. S2B). Our results suggest that GRAF1 mediates endocytic surface removal and inactivation of Rab8 in order to balance membrane tension at protrusions. In this way, endocytic turnover might regulate exocytic delivery and enrichment of polarity components between growing and retracting regions of the cell surface (Fig. 4E).

### GRAF1 depletion disturbs regulation of cell polarity and affects lumen formation

**Fig. 5.**
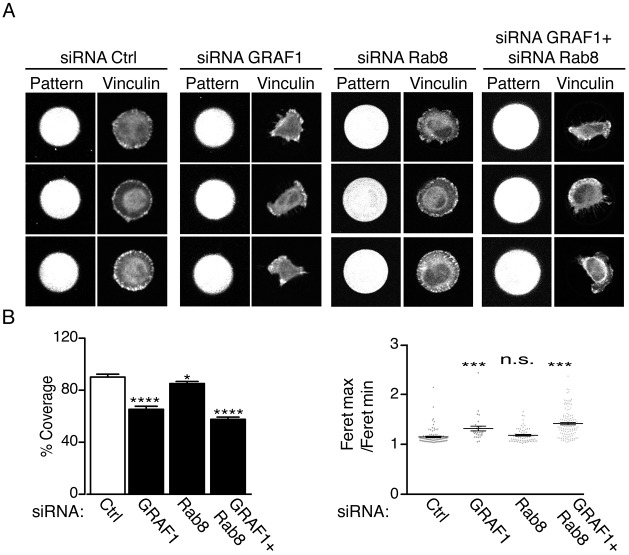
**GRAF1 depletion impairs the adjustment of polarization in HeLa cells during cell spreading.** (A) Representative micrographs showing the spreading abilities of GFP–GRAF1-expressing Flp-In T-REx HeLa cells transfected with indicated siRNA and seeded on micropatterns as described in the Materials and Methods. Cells were stained for vinculin to visualize cell morphology. (B) The left panel shows the quantification of percentage of total surface of the micropattern covered by cells. The right panel shows the ratio between the maximum and minimum Feret's radius; the higher the ratio is, the more elongated cells. Results are mean±s.d. for *n*=30 cells per condition from three independent experiments *P≤0.05; ***P≤0.001; ****P≤0.0001; n.s., not significant (Kruskall–Wallis test).

As previously shown, cells lacking GRAF1 displayed a fusiform morphology (Fig. S1H). The fusiform morphology and the polarized localization of Rab8, MT1-MMP and p-Par6, suggested that GRAF1 depletion might result in spontaneous polarization with stochastically determined orientation. To further study this, we used circular micropatterns to restrict the spreading of cells to this shape and size (Fig. 5A; Fig. S3A) (Thery, 2010). An analysis of the area covered by the cells clearly showed that cells lacking GRAF1 were not able to spread and cover the micropattern, unlike the control cells and cells depleted of Rab8 (Fig. 5A,B). Analysis of the Feret's diameter confirmed that cells became elongated when GRAF1 was depleted (Fig. 5B, right panel). The inability of GRAF1-depleted cells to form protrusions in all directions and their deficiency in multi-directional spreading is the opposite of the phenotype described for cells lacking Rab8 (Hattula et al., 2006). Our data suggest that GRAF1 plays an essential role for the adjustment of polarization in HeLa cells during cell spreading.

**Fig. 6.**
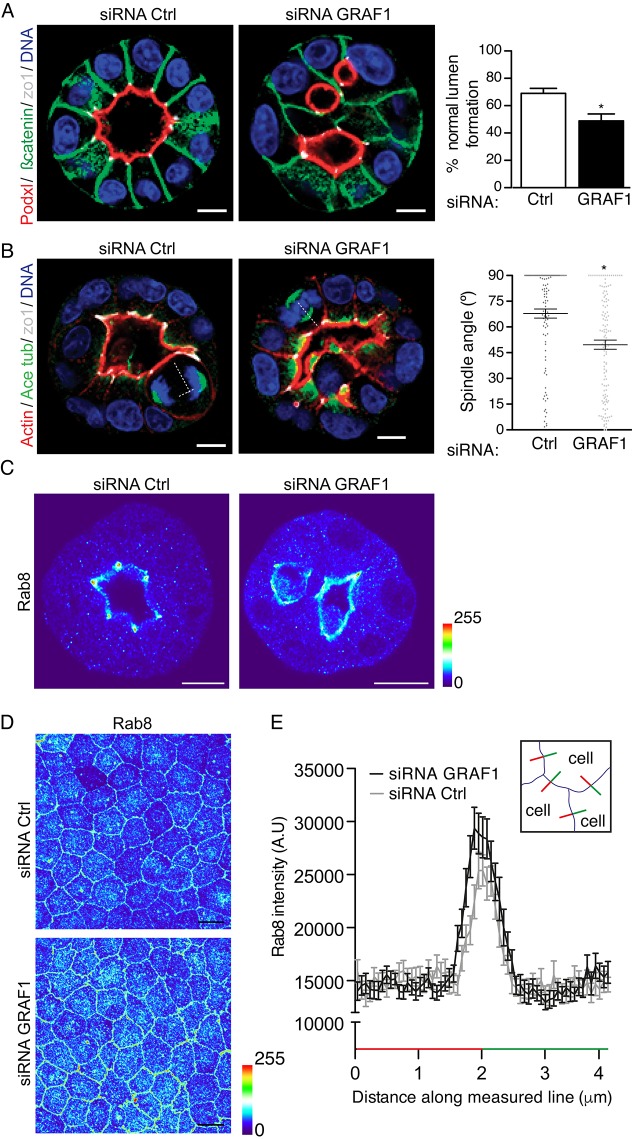
**GRAF1 depletion affects lumen formation and spindle orientation.** (A) Representative images of MDCK cells transfected with control siRNA (Ctrl) or siRNA against GRAF1 and subjected to 3D culture. The right panel shows the mean±s.e.m. percentage of normal lumen formation from three independent experiments. (B) Representative images of cysts showing the spindle angle in MDCK cells transfected with control siRNA (Ctrl) or siRNA against GRAF1. The right panel shows the mean±s.e.m. quantification of the spindle angle from three independent experiments. *P≤0.05 (*t*-test). Scale bars: 5 μm. (C) Representative micrographs of MDCK cells grown in 3D and stained with Rab8 antibody. (D) Representative micrographs of MDCK cells grown as a single monolayer in transwells to facilitate visualization and quantification of Rab8 at the apical membrane. Images show cells transfected with control siRNA (Ctrl) or siRNA against GRAF1 and the pseudo color represents localized fluorescence intensity. (E) Quantification of Rab8 intensity from D in cells treated as in C. Intensity was measured using ImageJ software along a 4 µm line centered over the plasma membranes of two opposing cells as illustrated in the cartoon inset. Results are mean±s.d. for 40 cells per condition. Scale bars: 10 μm.

To study whether GRAF1 might also be involved in the regulation of apical and basolateral polarity, we used Madin–Darby canine kidney (MDCK) cells. Immunoblotting and immunofluorescent analysis revealed that these cells expressed GRAF1, which was localized to the cell surface (Fig. S3B,C). Next, we addressed the role of GRAF1 in a model of epithelial morphogenesis. For that, MDCK cells were grown under 3D culturing conditions, where they assemble into organotypic epithelial entities that resemble *in vivo* physiological conditions (Shamir and Ewald, 2014). We observed that a 77% reduction in GRAF1 protein expression (Fig. S3C) was sufficient to alter normal lumen formation (Fig. 6A), and compromise normal spindle orientation during cell division (Fig. 6B). In addition, we observed that the localization of Rab8 was more homogeneously distributed throughout the apical membrane in the distorted cysts, and did not accumulate at the apical cell junctions to the same extent as in the control (Fig. 6C). To be able to measure this potential effect on Rab8 localization, MDCK cells were grown as a single epithelial monolayer in transwell chambers to induce apical and basolateral polarization. In this system, Rab8 was polarized to the apical membrane (Fig. 6D), as previously shown (Bryant et al., 2010). The intensity of the Rab8 staining in multiple cells was quantified along a 4 µm line centered over the plasma membranes of two opposing cells. This analysis showed that the accumulation of Rab8 at the membrane was increased in GRAF1-depleted cells compared to control (Fig. 6E). Previous studies have shown that both Rab8 and Cdc42 are necessary for normal lumen formation in the 3D MDCK model (Bryant et al., 2010; Gálvez-Santisteban et al., 2012; Martin-Belmonte et al., 2007). Our results suggest that GRAF1 might also be involved in epithelial morphogenesis, although the mechanism by which GRAF1 influences lumen formation and Rab8 localization in these cells is still elusive.

Taken together, our data show that GRAF1 activity is important for surface removal and inactivation of Rab8, which counterbalances exocytosis and regulates the local activity of Cdc42 and Rab8 during polarity adjustments.

## DISCUSSION

The compensatory membrane redistribution that accounts for volume changes during the undulating growth and retraction of cellular protrusions is thought to depend on a dynamic balance between exocytosis and endocytosis. These processes will buffer membrane tension and modify the local protein and lipid composition to adjust or maintain polarity of protrusions (Houk et al., 2012; Raucher and Sheetz, 2000; Schmoranzer et al., 2003). Here, we demonstrate that the molecular components that promote exocytosis and endocytosis, respectively, are directly coupled to facilitate cross-regulation between these two processes. We show that the activity of the key exocytic component, Rab8, is counterbalanced by surface removal and inactivation via the CLIC endocytic pathway at cellular protrusions. GTP-bound Rab8 is removed by incorporation into vesicles and tubules coated by GRAF1, which together with Cdc42, acts as a positive regulator of the CLIC pathway. This high-capacity pathway is known to build up a large pool of internal membranes (Howes et al., 2010). Our data suggest that Rab8 is not required for the generation of GRAF1-coated CLICs. Instead, we propose that Rab8 should be considered as a cargo of CLICs. By altering the osmolarity of the medium surrounding the cells, we mimicked the dynamic changes in membrane tension that take place at protrusions. These experiments showed that Rab8 was recruited to the cell surface following tension increase, in line with its exocytic role. Interestingly, following an acute decrease in membrane tension, GRAF1 rapidly accumulated in discrete punctate and vesicular structures at the cell surface. Our data indicate that the endocytic activity of GRAF1, together with the fusogenic activity of GTP-bound Rab8, could function as a regulatory feedback mechanism to balance the cell surface area through the expansion or recycling of the internal pool of membranes.

We found that GRAF1 associated with the active form of Rab8 and that GRAF1-depletion resulted in elevated levels of GTP-bound Rab8. These data are in agreement with a previous study showing that that endocytosis stimulates the GTP-hydrolysis of Rab8 (Vaibhava et al., 2012), and that membrane trafficking is important for Rab8 to meet its GEFs and GAPs (Hattula et al., 2006; Hokanson and Bretscher, 2012; Vaibhava et al., 2012). GRAF1 does not, however, contain a typical Rab GAP domain and we were not able to detect any interaction between GRAF1 and Rab8, suggesting that GRAF1 does not directly activate GTP hydrolysis by Rab8. Instead, we propose that the spatial segregation of Rab8 into GRAF1-positive membrane carriers might promote the interaction with a different GAP. Using an APEX-based biotinylation approach, we were able to show that Rab8, together with MICAL-L1 and EHD1, were localized in the near proximity of GRAF1 in cells following expression of Cdc42Q61L. These data suggest that several proteins might be involved in the surface removal and inactivation of Rab8. MICAL-L1 has been shown to bind to both GRAF1 and Rab8, and might facilitate the coupling between GRAF1 and Rab8 on endocytic membranes. Interestingly, we also found that the increased Rab8 activity following GRAF1 depletion resulted in an accumulation of surface-associated Rab8 and Rab8-positive vesicles containing the exocytic cargo MT1-MMP at the protrusions. Imaging analysis revealed that some of these vesicles fused with the plasma membrane while others appeared to be stalled. There are several mechanistic explanations that could account for the stalled vesicles: (1) the locally increased Rab8 activity at the surface might prevent fusion; (2) the fusing machinery might be sequestered due to the local increase in vesicles; or (3) GRAF1 depletion could result in decreased membrane tension due to reduced endocytosis, thus hampering vesicle fusion. In addition, we observed that the growth of the protrusions appeared to be hindered in GRAF1-depleted cells, suggesting that the trafficking defect impaired the protrusion dynamics.

Previous work has shown that GRAF1 knockdown results in cell elongation and impaired cell spreading (Doherty et al., 2011; Simpson et al., 2012). Based on our data, we propose that this is due to an inability of these cells to sense and adjust the polarity axis and direction of spreading. We found that HeLa cells depleted of GRAF1 were not able to adapt their spreading to circular micropatterns. Instead, these cells appeared to adopt a spontaneous polarization with stochastically determined orientation. This defect in multidimensional spreading might be due to the inability to control membrane tension and adjust the redistribution of membranes and key components so that polarity can be modified. We found that in cells lacking GRAF1, the otherwise uniform plasma membrane localization of p-Par6 was highly polarized. In addition, the total amount of phosphorylated PKCζ was reduced by 50%. Autophosphorylation of PKCζ and phosphorylation of Par6 are mediated by Cdc42. Therefore, our data suggest that the local Cdc42 activity was compromised following GRAF1 depletion. However, we did not detect any difference in the total GTP-bound Cdc42 in such cells.

Rab8, together with Cdc42, has previously been shown to be essential for maintaining epithelial polarity and intestinal integrity and physiology *in vivo* (Bryant et al., 2010; Sakamori et al., 2012; Sato et al., 2007). Interestingly, a recent study showed that recurrent fusion mutations in gastric cancer involving GRAF1 resulted in the loss of epithelial integrity and induced an epithelial-to-mesenchymal transition (Yao et al., 2015). When we reduced the GRAF1 levels in 3D-cultured MDCK cells, a significant impairment of lumen formation was observed, showing that GRAF1 also influences epithelial polarization. We could furthermore show that a reduction in GRAF1 levels affected spindle orientation during cell division. Silencing of Cdc42 in the 3D MDCK model has previously been shown to generate defects in endocytic and exocytic vesicle trafficking, and to compromise the correct orientation of the mitotic spindle during cell division (Harris and Tepass, 2010; Jaffe et al., 2008; Martin-Belmonte et al., 2007). Furthermore, Rab8 has been described to mediate the vesicular trafficking of Cdc42 to the apical surface together with Par6 and aPKC (Bryant et al., 2010). We found that GRAF1 depletion altered the apical localization of Rab8 in the MDCK cells,; we were not, however, able to verify the surface removal of Rab8 via GRAF1, as was found in HeLa cells. Our data suggests that GRAF1 is involved in the regulation of epithelial cell polarity, but the mechanism is still to be determined.

In conclusion, we propose that endocytic turnover and inactivation of Rab8 and Cdc42 mediated by GRAF1-mediated endocytosis is important for balancing membrane redistribution between growing and retracting regions of the cell. Impairment of this process results in an inability to adjust the polarity axis.

## MATERIALS AND METHODS

### Constructs, antibodies and reagents

DsRed–Rab7a and DsRed–Rab11a (Addgene), mCherry-tagged Rab8aWT, Rab8aQ67L, Rab8aT22N, and MICAL-L1-CT together with GST–JCF1D1 were as previously described (Hattula et al., 2002, 2006). MT1MMP–mRFP was kindly provided by María C. Montoya [Cellomics Spanish National Center for Cardiovascular Research (CNIC), Madrid, Spain]. pTagBFP-PH-FAPP1 was obtained by subcloning GST–PH-FAPP1 (Hammond et al., 2009; kindly provided by Gerald R.V. Hammond, Dept. Cell Biology University of Pittsburg, USA) in the pTagBFP-C1 (Evrogen) using EcoR1 and Sal1 restriction enzymes. pTagBFP-Rab5a and Myc-Cdc42Q61L were as previously described (Francis et al., 2015). 10,000 Da Dextran conjugated to Alexa Fluor 555 or FITC, and CTxB conjugated to Alexa Fluor 647 were from Molecular Probes. Antibodies used were: goat anti-aldolase [western blotting (WB) 1:5000; AB1809, Chemicon International], mouse anti-GAPDH (WB 1:1000; #MAB374, Millipore), rabbit anti-Cdc42 (WB 1:1000), rabbit anti-EHD1 (WB 1:1000) and mouse anti-vinculin [immunofluorescence (IF) 1:100; ab109553, ab109747 and hVIN-1 ab1194, Abcam], rabbit anti phospho-PKCζ/λ and PKCζ total (both WB 1:1000, 9378 and 9368, Cell Signaling), rabbit anti-Rab11 (WB 1:1000; 71-5300, Invitrogen), mouse anti-MICAL-L1 (WB 1:1000; H00085377-B01P, Novus Biologicals), streptavidin–HRP (WB 1:100,000; 21130, Thermo Scientific), mouse anti-clathrin heavy chain (WB 1:1000) and mouse anti-Rab8 (WB 1:1000; clone 23 610499, 610844, BD Biosciences), rabbit anti-Rab8 (IF 1:100; Hattula et al., 2002), mouse and rabbit anti-GRAF1 RA-83 (WB 1:1000), produced as previously described (Lundmark et al., 2008), and anti-p-Par6 (IF 1:100) was kindly provided by Maréne Landström (Dept. of Medical Biosciences, University of Umeå, Umeå, Sweden). Secondary antibodies were conjugated to horseradish peroxidase (HRP; Sigma-Aldrich and Agrisera) and IRDye 800CW or 680RD (LI-COR bioscience).

### Cell lines

Rab8a and the isoform GRAF1b were used in the constructed cell lines. Constructs for bistronic and inducible expression of GFP–GRAF1 and mCherry–Rab8 was generated by cloning the DNA encoding the P2A peptide with a flanking mCherry tag from the pSYC-97 vector (Kim, 2011) (a kind gift from Seok-Yong Choi, Department of Biomedical Sciences, Chonnam National University Medical School, Gwangju, Republic of Korea) sequentially into pcDNA5/FRT/TO/GFP-GRAF1 with Rab8 to obtain the construct pcDNA5/FRT/TO/eGFP-GRAF1-P2A-mCherry-Rab8. This construct was modified by PCR mutagenesis to generate a construct for double expression of GFP–GRAF1 and mCherry-Rab8Q67L using the forward primer 5′-GACACAGCCGGTCTGGAACGGTTTCGGACGATCACAAC-3′ and the reverse primer 5′-CCGAAACCGTTCCAGACCGGCTGTGTCCCATATCTGCA-3′ to obtain the construct pcDNA5/FRT/TO/eGFP-GRAF1-P2A-mCherry-Rab8Q67L. The pcDNA/FRT/TO/mCherry-Rab8 construct was generated by PCR cloning on pcDNA5/FRT/TO/eGFP-GRAF1-P2A-mCherry-Rab8. Similarly, we generated a pcDNA5/FRT/TO/GRAF1-APEX2 cell line by PCR cloning the APEX2 DNA into the pcDNA5/FRT/TO/GRAF1 construct. APEX was PCR cloned from pcDNA3-mito-APEX (Addgene plasmid # 42607, kindly deposited by Alice Ting), which was mutated into APEX2 according to Alice Ting's directions.

### Cell culture and transfection

HeLa cells (ATCC-CRM-CCL-2) were cultured in DMEM (high glucose, L-glutamine, sodium pyruvate, HEPES and Phenol Red), supplemented with 10% fetal bovine serum (Gibco). For stable cell lines, the culturing medium was further supplemented with 100 μg/ml hygromycin B and 5 μg/ml blasticidin S HCl (Invitrogen) for plasmid selection, and recombinant protein expression was induced by incubation in 1 ng/ml doxycycline hyclate (Sigma-Aldrich) for 24 h. MDCKII cells were cultured in complete MEM supplemented with 5% fetal bovine serum, 50 U/ml penicillin and 50 μg/ml streptomycin. Cyst culture was performed as described previously (Rodriguez-Fraticelli et al., 2010). For separate access to apical or basolateral domains, MDCK cells were seeded at confluent levels on 24 mm polyester tissue culture inserts of 0.4 μm pore size (Transwell; Costar Inc.). Targeted protein silencing was accomplished with siRNA against GRAF1 (‘siRNAb’; Stealth RNAi, Invitrogen), siRNA against Cdc42 (ON-TARGETplus siRNA, Dharmacon) (Lundmark et al., 2008), and against Rab8a (HSS106459, HSS181075, HSS181076; stealth RNAi, Invitrogen), Medium GC Duplex RNA (Stealth RNAi, Invitrogen) served as the negative control. Transfections for transient expression of plasmids (24 h) and siRNA (72 h) were performed using Lipofectamine 2000 (Invitrogen) according to the manufacturer's recommendations. Protein expression levels were analyzed in cleared cell lysates by western blotting using antigen detection and an Odyssey Sa reader (LI-COR Biosciences).

### Biotin-phenol labeling and biotinylated protein pulldown

GRAF1-APEX2 Flp-In TRex HeLa cells were transfected with pCMV-myc-cdc42Q61L plasmids as described above. After 24 h the cells were labeled with 500 µM of biotin-phenol at 37°C for 30 min, as described previously (Hung et al., 2016) except that the cells were incubated in 1 mM H_2_O_2_ at room temperature for 10 min, with occasional shaking. The cells were washed three times with quenching buffer (PBS containing 5 mM Trolox, 10 mM sodium azide, and 10 mM sodium ascorbate) to stop the labeling reaction. The cells were scraped and lysed with RIPA lysis buffer (25 mM HEPES pH 7.5, 150 mM NaCl, 1% NP-40, 1% sodium deoxycholate, 0.1% SDS, and protease inhibitor cocktails) for 30 min on ice. Cleared lysate were mixed with neutravidin high-capacity agarose beads (Thermo Scientific) overnight at 4°C. The neutravidin beads were washed five times with RIPA lysis buffer followed by two washes with SDS washing buffer (100 mM Tris-HCl pH 6.8, 4% SDS, 10 mM DTT). Biotinylated proteins were eluted by resuspending the beads in 2× SDS loading buffer and heating at 75°C for 10 min. Biotin-phenol labeling was evaluated by western blotting. Elution from neutravidin beads was through boiling at 95°C for 10 min, and then separation by 10% SDS-PAGE.

### Cdc42 activity pulldown

The Cdc42 activity pulldown assay was performed in GFP-GRAF1 FLPin T-REx Hela cells transfected with the indicated siRNAs. The pulldown was performed using the Cdc42 pulldown activation assay biochemistry kit (bead pull-down format; cat. # BK034-S) from Cytoskeleton following the manufacturer's instructions.

### Time-lapse microscopy, confocal microscopy and dextran-uptake experiments

Cells were seeded and induced with 1 ng/ml doxycyclin on 25 mm^2^ coverslips at 24 h prior to imaging. Before imaging, the coverslip was transferred to the Attofluor^®^ cell chamber (Invitrogen) and culture medium was replaced with live-cell high-glucose DMEM with HEPES and without Phenol Red supplemented with pyruvate (Gibco). Live-cell imaging was performed under controlled conditions of CO_2_ (5%) and temperature (37°C) with a 63× lens (Plan-Apochromat 1.40 Oil DIC M27) Zeiss Cell Observer Spinning Disk Confocal controlled by ZEN interface with an Axio Observer.Z1 inverted microscope, equipped with a CSU-X1A 5000 Spinning Disk Unit and a EMCCD camera iXon Ultra from ANDOR. The dextran uptake was performed at 37°C for 5 min in the live-cell chamber of the microscope, then the cells were washed twice with warm medium and the imaging was started as quickly as possible. For fixed cells, after dextran uptake cells were washed twice and then fixed according to Llado et al. (2004). Immunofluorescence was performed as previously described in (Lundmark et al., 2008). Confocal images of fixed cells were acquired with a Nikon A1R confocal (LSM) controlled by Nikon NIS elements interface with a Nikon Eclipse Ti-E inverted microscope equipped with CFI Plan Apochromat 60× oil (N.A. 1.40). Confocal images of fixed cysts were acquired using an Axio Observer.Z1 inverted microscope equipped with LSM 880 with Airyscan detector controlled by Zeiss Zen Black, 63x lens (Plan-Apochromat 1.40 Oil DIC M27).

### Image analysis

Tracking of structures positive for GFP-GRAF1 and mCherry-Rab8 was performed using Imaris software. We made a *region of interest* (ROI) for the protrusion, then GFP-GRAF1 and mCherry-Rab8 were segmented as spots. We chose structures with an estimated diameter of 0.8 μm, applied background subtraction and the algorithm that was used was autoregressive motion with Max distance=1 μm and Max gap size=3. Only tracks with duration above 3 s are shown. Structures positive for GRAF1 and Rab8 that appeared at growing protrusions were classified into 4 categories and mCherry-Rab8 channel was used as reference channel. If GFP-GRAF1 was present in the same structure with mCherry-Rab8 within the first 5 frames we counted this as “GRAF1 in the beginning”, on the other hand, if GFP-GRAF1 appeared 5 frames before mCherry-Rab8 disappeared, we counted as “GRAF1 at the end”. mCherry-Rab8 structures negative for GRAF1 were included in the “No GRAF1” category. Micrographs and acquired movies were prepared (cropped, rotated, linearly adjusted for intensity and converted) using ImageJ. To quantify the overlap between GRAF1 and different Rabs, images acquired by spinning disk microscopy were analyzed as follows. The GRAF1 and Rab channels were segmented using the Feature extraction Laplacian plugin in ImageJ. GRAF1-positive structures that colocalized with the Rab-positive structures were counted and represented as the percentage of GRAF1 structures positive for Rab. Quantification of Rab8 intensity in MDCK cells was performed using Image J software. Intensity was measured along a 4 µm line centered over the plasma membranes of two opposing cells. 40 cells were analyzed per condition.

### Recovery experiment

CellASIC^®^ ONIX microfluidics system (Millipore) was used for experiments where cell culture medium was exchanged. The microfluidic plate (Millipore cat. no. M04S-03) was loaded with induced double Flp-In T-REx HeLa cells according to the manufacturer's instructions 24 h before the experiment. On the day of the imaging, the plate was attached to the stage of the microscope. Medium exchange was performed at 5 psi (34.5 kPa) for 1 min followed by constant flow at 0.25 psi (1.7 kPa) both for water addition (hypotonic treatment) and for medium add-back (recovery). To quantify the amount of Rab8 at the plasma membrane versus that in cytosol, a line was drawn on the Rab8 channel. The line was perpendicular to the membrane where the center of the line corresponded to the plasma membrane and one end of the line was in the cytosol and the other end was outside the cell. The intensity value at the center of the line was divided by the intensity value at the cytosol. The number of colocalized spots during the recovery experiment was obtained by segmenting the whole image and both channels as spots, as described above. The recruitment analysis was performed by visual assessment; the time between the VLD appearance and Rab8 or GRAF1 appearance was then plotted as recruitment to VLDs.

### Spreading assay

HeLa Flp-In T-REx expressing GFP-GRAF1 were transfected with siRNA for 72 h as described above. Cells were seeded on fibronectin-650-coated CYTOOchips™ with a circular pattern (DC-S-A X18) according to the manufacturer's instructions. The cells were allowed to spread for 3 h and then fixed and stained with anti-vinculin antibody as described above. The confocal plane in which the cells had the biggest surface was used to quantify the percentage of coverage of the micropattern. This was performed using the area measurement tool in Nis Elements software; this tool also provided the Feret's diameters maximum and minimum.

### Statistics

Statistical tests were performed using Prism 5 (GraphPad Software) with the indicated sample size and number of independent experiments. All quantifications are visualized as the mean±s.d. unless otherwise stated. *P≤0.05; **P≤0.01; ***P≤0.001; ****P≤0.0001; N.S. not significant.
